# Development of Retinal Infarct Due to Intracameral Cefuroxime Injection Following Complicated Cataract Surgery

**DOI:** 10.4274/tjo.61580

**Published:** 2018-12-27

**Authors:** Sabahattin Sül, Aylin Karalezli

**Affiliations:** 1 Muğla Sıtkı Koçman University Faculty of Medicine, Department of Ophthalmology, Muğla, Turkey

**Keywords:** Intracameral cefuroxime, retinal toxicity, retinal infarct

## Abstract

We present the case of a 60-year-old patient who underwent a complicated cataract surgery with cefuroxime injection (1 mg/0.1 mL) into the anterior chamber at the end of surgery. The patient presented to our hospital due to decrease in visual acuity (VA) after surgery. VA was counting fingers (CF) from 4 meters. There was extensive retinal hemorrhages and edema in addition to retinal vascular leakage detected with fluorescein angiography (FA). After negative microbiologic tests, the patient was treated with intravenous pulse and oral corticosteroids. Rheumatologic investigation was also negative. At month 5, VA was CF from 1 meter in addition to disseminated capillary loss in FA and optic nerve atrophy despite corticosteroid treatment. The patient developed retinal infarction due to cefuroxime injection following a complicated cataract surgery. Surgeons and surgical staff should be aware of the possibility of retinal toxicity while using cefuroxime, particularly in complicated cases.

## Introduction

Bacterial endophthalmitis is the most feared complication of cataract surgery and can cause severe and permanent visual loss.^1^ Intracameral antibiotic injection has decreased the incidence of postoperative endophthalmitis.^2^ Cefuroxime, moxifloxacin and vancomycin are the preferred antibiotics for cataract surgery.^3,4,5^ Cefuroxime has been reported to provide a five-fold decrease in endophthalmitis incidence.^6^ A concentration of 1 mg/0.1 mL is the recommended dose for microbial efficacy and tissue safety.^2^ However, in the absence of ready-to-use formulations, dilution errors may be overlooked while preparing the desired concentration. Exposure to high-dose cefuroxime can cause retinal toxicity, which can result in retinal and optic nerve infarct.^7^ The retinas become more sensitive to the drug doses due to disruption of the barriers between the anterior and posterior segments in complicated surgeries.

## Case Report

**Figure 1:**
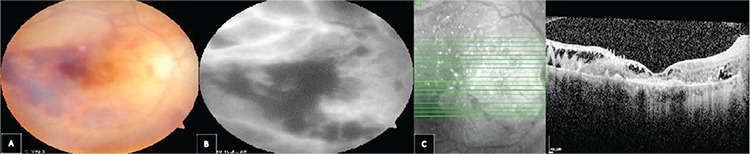
A) Fundus photography of the patient shows retinal hemorrhages particularly at the central retina. B) Fluorescein angiography shows vascular leakage. C) Foveal thinning and outer segment atrophy in optical coherence tomography

**Figure 2:**
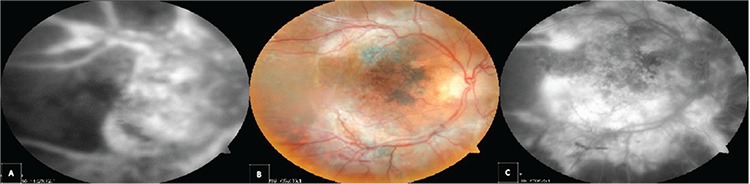
A) At 1 month, hemorrhages were substantially resolved but there was persistent severe vascular leakage and capillary loss in fluorescein angiography despite high-dose anti-inflammatory treatment, B) Fundus photography at 5 months shows neovascular membrane formation, C) Fluorescein angiography shows minimal vascular leakage and extensive retinal infarction

A 60-year-old patient presented to our hospital due to decreased vision following cataract surgery. The patient underwent a complicated cataract surgery (posterior capsule rupture and anterior vitrectomy) with implantation of a 3-piece foldable IOL in the sulcus and a recommended dose (1 mg/0.1 mL) of cefuroxime was injected into the anterior chamber. Visual acuity (VA) was counting fingers from 4 meters. There were +2 cells in the vitreous, retinal hemorrhages and edema, particularly at the posterior pole (Figure 1A). Fluorescein angiography (FA) revealed extensive vascular leakage (Figure 1B). Foveal thinning and outer segment atrophy were observed in optical coherence tomography (Figure 1C). Microbiologic tests (viral and parasitic antibodies and polymerase chain reaction [PCR] analysis of vitreous samples) were negative. Treatment with 1000 mg intravenous pulse corticosteroid was initiated and continued for 3 days. Medical treatment continued with 1 mg/kg oral corticosteroid for 1 month. Meanwhile, rheumatologic etiologies, which can cause retinal vasculitis, were investigated but the results were negative. After 1 month, the retinal hemorrhages had substantially regressed but there were persistent vascular leakage and retinal capillary loss (Figure 2A). At 5 months, VA decreased to counting fingers from 1 meter. Corneal edema, anterior chamber and vitreous cells, and retinal hemorrhages resolved, but the optic nerve was pale and retinal neovascularization developed (Figure 2B). FA showed minimal vascular leakage in addition to extensive retinal infarct (Figure 2C).

## Discussion

The patient in this report presented with vitritis, retinal hemorrhages, vascular leakage, and capillary infarct in FA, which were suggestive of obstructive retinal vasculitis due to rheumatologic diseases or viral retinitis. However, the patient did not have a rheumatologic disease history and clinical investigation for rheumatologic diseases (Behçet’s disease, systemic lupus erythematosus, inflammatory bowel disease, polyarthritis, multiple sclerosis, sarcoidosis, etc.) was negative. Viral antibodies (particularly to herpes simplex, varicella zoster, or cytomegalovirus) and PCR analysis were also negative.

Aprokam is the ready-to-use formulation of cefuroxime; however, in the absence of the commercial formulation, the recommended cefuroxime concentration is prepared with the surgeons’ own dilution procedures. Although the recommended drug concentration can be prepared properly with these procedures, it is nevertheless possible for the surgeon or other personnel to make a mistake during dilution, as shown by previous reports. Çiftçi et al.^7^ reported 50 to 70 mg, Qureshi and Clark reported 62.5 mg, Delyfer et al. reported 40 to 50 mg and Olavi reported 10 to 100 mg cefuroxime exposure at the end of surgery.^8,9,10^

Cefuroxime toxicity varies from case to case and the severity of its clinical manifestations is associated with surgical complications as well as drug concentration. In uncomplicated cases, a mild, transient, and reversible retinal toxicity may occur with the recommended dose injection, whereas high-dose exposure can cause severe complications such as macular infarction.^8,11^ Furthermore, in complicated cases, more severe complications characterized by extensive retinal edema, hemorrhage, disseminated capillary loss, and optic nerve atrophy can develop after cefuroxime injection.^7^ This is due to the absence of a lens capsule barrier limiting the passage of the drug to the posterior segment in complicated cases. The severity of the clinical features in the present case may be associated with posterior capsule rupture, direct retinal exposure to the drug, or breakdown of the blood-retinal barrier due to drug toxicity. Extensive retinal capillary loss and optic atrophy were signs of the retinal and optic nerve infarction, which was previously demonstrated by Çiftci et al.^7^ Pars plana vitrectomy might also be considered together with anti-inflammatory treatment to minimize retinal exposure to the toxic agent, particularly in severe cases. In addition, retinal tears and retinal detachment may develop due to retinal infarction. For that reason, patients should be followed very closely and argon laser photocoagulation should be considered in these cases during the follow-up period if needed. In our patient, the clinical course did not respond to intensive anti-inflammatory treatment. Therefore, surgeons and staff should adjust the intracameral drug dose accordingly in complicated cataract surgeries to prevent the development of severe complications related to drug toxicity.

In conclusion, retinal toxicity may develop in complicated cases with the recommended cefuroxime concentration. Visual outcome seems to be poor despite high dose anti-inflammatory treatment.
